# A State‐Gated Metallo‐DNAzyme Framework Programs Tumor Stress Vulnerability Through Metabolic Licensing

**DOI:** 10.1002/advs.77520

**Published:** 2026-09-08

**Authors:** Xiaodong Ma, Lingyun He, Xinru Wang, Yuanyuan Wei, Chengcheng Li, Jiaqi Yan, Weijian Sun, Xian Shen, Hongbo Zhang

**Affiliations:** ^1^ NHC Key Laboratory of Clinical Nutrition and Intervention The First Affiliated Hospital of Wenzhou Medical University Wenzhou China; ^2^ Pharmaceutical Sciences Laboratory Faculty of Science and Engineering Åbo Akademi University Turku Finland; ^3^ Zhejiang Key Laboratory of Intelligent Cancer Biomarker Discovery and Translation The First Affiliated Hospital of Wenzhou Medical University Wenzhou China; ^4^ Turku Bioscience Centre University of Turku and Åbo Akademi University Turku Finland

**Keywords:** metabolic licensing, Metallo‐DNAzyme framework, programmable stress vulnerability, state‐gated therapeutic materials, tumor metabolic buffering

## Abstract

Stress‐generating therapeutic materials are commonly optimized to deliver cytotoxic inputs, but delivery alone does not determine whether intracellular stress becomes irreversible damage. Tumor cells can buffer metal‐, redox‐ and mitochondrial stress through glucose‐dependent metabolism, creating a gap between stress exposure and stress execution. Here, we report a tumor‐state‐gated metallo‐DNAzyme framework, C@HGDz^2^ ^1^/CaCu, that introduces metabolic licensing as a programmable material function. The framework couples a miR‐21/APE1‐gated GLUT1 DNAzyme with a Ca/Cu‐partitioned metal–nucleic‐acid architecture and co‐assembled cystine. Tumor‐state logic confines GLUT1 mRNA cleavage to dual‐input cancer‐associated conditions, whereas metal partitioning assigns Ca^2^^+^ to DNAzyme‐compatible catalysis and Cu^2^^+^ to framework persistence and copper‐associated stress within the metabolically rewired cellular state. After HER2‐guided deployment, C@HGDz^2^ ^1^/CaCu suppresses GLUT1 and glycolytic signaling, restores PTEN‐associated signaling and remodels glucose‐derived metabolic buffering. This metabolic remodeling is associated with enhanced engagement of copper‐ and cystine‐dependent oxidative, mitochondrial and cytoskeletal stress. In an orthotopic gastric tumor model, the framework enhances tumor accumulation, shows coordinated metabolic rewiring and stress engagement, and suppresses tumor progression with apparent tolerability under the tested regimen. This work establishes state‐gated metallo‐DNAzyme framework engineering as a strategy for shifting stress‐generating materials from cytotoxic delivery to vulnerability programming.

## Graphical abstract

A tumor‐state‐gated metallo‐DNAzyme framework integrates miR‐21/APE1‐responsive GLUT1 silencing with Ca/Cu‐partitioned metal–nucleic acid assembly and cystine loading. The framework first weakens glucose‐dependent metabolic buffering and then amplifies copper‐ and disulfide‐associated stress, converting tumor metabolic adaptation into programmable vulnerability and suppressing orthotopic gastric tumor progression after systemic administration.

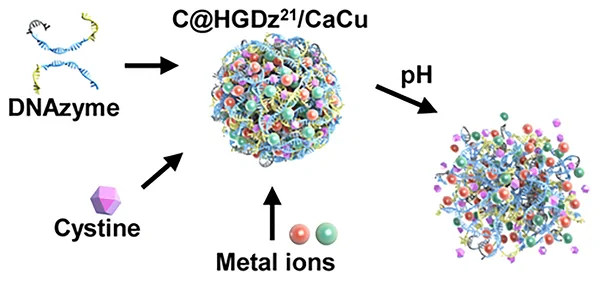

## 1 Introduction

For stress‐generating therapeutic materials, delivery is not equivalent to damage execution. A material may accumulate in tumors and release cytotoxic inputs inside cancer cells, yet the biological outcome still depends on whether those cells are in a state that permits the released stress to become irreversible damage. In solid tumors, oxidative pressure, mitochondrial perturbation, metal‐associated proteotoxic stress and redox‐disruptive cues can be absorbed by metabolic flux, adaptive signaling and stress‐handling capacity before they cross a damage‐execution threshold [1, 2, 3, 4, 5, 6, 7, 8]. This creates a central design problem for therapeutic materials. Increasing the amount of stress delivered is unlikely to be sufficient if tumor cells remain able to buffer and recover from that stress. Effective stress‐generating materials should therefore not only deliver cytotoxic inputs, but also weaken the intracellular buffering networks that prevent those inputs from becoming biologically executable.

Tumor metabolism offers an actionable route to this form of state‐controlled stress execution. Glycolysis‐dominated metabolism is widely recognized as an energetic and biosynthetic programme, but in therapeutically challenged tumor cells it also acts as a stress‐buffering system [9, 10, 11, 12, 13, 14]. Sustained glucose uptake supports glycolytic ATP production, while glucose‐derived flux through the pentose phosphate pathway supplies NADPH‐dependent redox protection [15, 16, 17, 18, 19, 20, 21, 22]. These outputs contribute to cellular energy and redox buffering under therapeutic pressure. Their loss may influence different stress modalities through distinct mechanisms, rather than generating a single uniform vulnerability state. As a result, metal‐ and disulfide‐associated stress inputs may remain only partially effective when they are released into a metabolically protected tumor‐cell state [23, 24, 25, 26, 27, 28, 29, 30, 31, 32]. A more selective strategy is therefore to incorporate metabolic licensing into material design, so that metabolic regulation weakens the cellular conditions that support stress tolerance and places co‐delivered stress inputs within a less protected tumor‐cell state.

GLUT1 provides a concrete node for this strategy because it links glucose entry to metabolic buffering and stress tolerance. By controlling glucose influx, GLUT1 supports glycolytic flux, energy homeostasis and redox protection [33, 34, 35, 36]. However, GLUT1 suppression alone may generate only transient vulnerability, because tumor cells can activate compensatory survival programmes after metabolic perturbation. microRNA‐21 (miR‐21), which is frequently upregulated in solid tumors, contributes to malignant survival and metabolic adaptation partly through PTEN repression and downstream PI3K/AKT/mTOR signaling [37, 38]. Coordinated regulation of GLUT1 and miR‐21 therefore provides a more integrated way to reprogramme the tumor metabolic state. It restricts glucose‐entry‐associated buffering while attenuating an adaptive oncogenic signaling axis. In this setting, GLUT1 regulation is not simply a metabolic inhibition strategy, but a state‐preparatory intervention that makes tumor cells more susceptible to subsequent metal‐ and disulfide‐stress engagement.

The challenge, therefore, is not merely to place a metabolic regulator and stress‐producing components in the same formulation. It is to construct a material architecture that functionally couples tumor‐state recognition, metabolic regulation and stress engagement within the same tumor cell. Separate interventions may provide these functions individually, but they do not readily integrate state recognition and stress‐conditioning within the same material architecture. Nucleic‐acid materials provide a unique basis for such sequence control because molecular recognition, catalytic gene regulation and framework assembly can be encoded into the same programmable structure [39, 40]. However, translating this logic into a metal–nucleic acid framework creates a specific materials‐level conflict. A metal environment compatible with DNAzyme folding and RNA cleavage is required for the metabolic‐licensing step, whereas a framework intended to persist during delivery and later supply metal‐associated stress requires a more durable and biologically active coordination component. Copper closes this design loop. By complementing the catalytic metal, copper reinforces the framework during deployment and subsequently provides a stress‐relevant source after glucose‐dependent buffering has been weakened. Thus, Ca/Cu functional partitioning provides a sequence‐organizing material principle that links framework persistence, metabolic licensing and post‐licensing stress execution within a single therapeutic sequence.

Here, we report a tumor‐state‐gated metallo‐DNAzyme framework, C@HGDz^2^ ^1^/CaCu, that combines sequence‐encoded molecular logic with metal‐ion functional partitioning. In the logic module, a GLUT1‐targeting DNAzyme is split into left and right partzymes, and the right partzyme is sequestered within an APE1‐responsive hairpin. miR‐21 promotes assembly of the catalytically competent complex and also serves as a tumor‐associated regulatory input, while APE1‐mediated unlocking provides a second endogenous activation requirement. This design was intended to restrict productive GLUT1 substrate cleavage to the combined presence of miR‐21 and APE1. Its dual‐input‐dependent catalytic behavior was evaluated under cell‐free conditions. HER2 aptamer modification further adds a spatial recognition layer for HER2‐expressing tumor cells.

**SCHEME 1:**
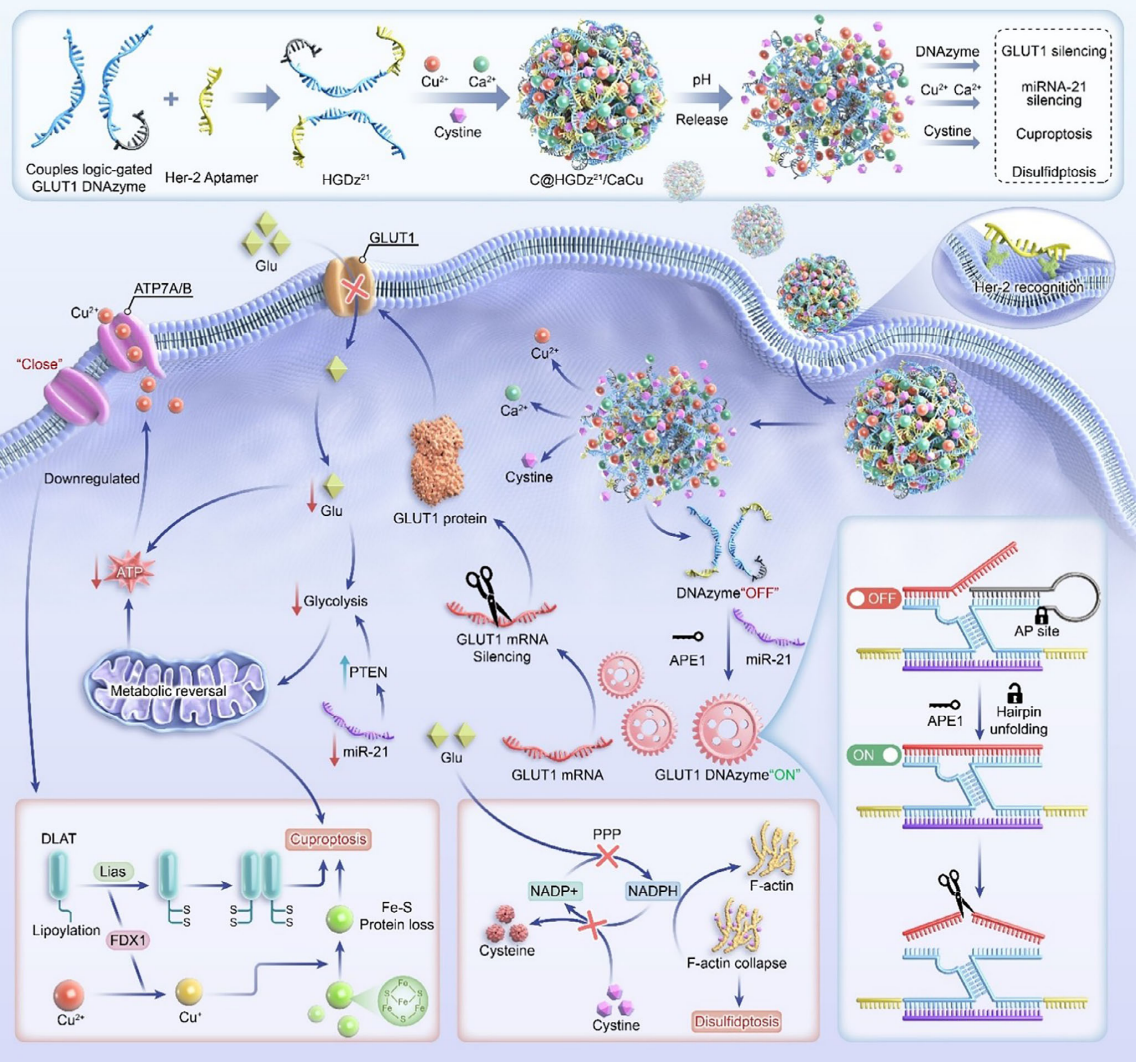
Schematic illustration of the construction and mechanism of the logic‐gated DNAzyme metal–nucleic acid framework. The upper panel shows the design and construction of the logic‐gated DNAzyme–MNF platform. Two DNA strands incorporating a HER2 aptamer, a GLUT1‐targeting DNAzyme motif, a miR‐21‐responsive module and an APE1‐responsive cleavage module are assembled into a tumor‐state‐gated DNAzyme architecture. Coordination with Ca^2^^+^ supports DNAzyme‐compatible folding and catalytic competence, whereas subsequent Cu^2^^+^ coordination reinforces the metal–nucleic acid framework and provides a copper‐associated stress source. Cystine is co‐assembled into the framework to introduce a disulfide‐stress input. The lower panel illustrates the proposed intracellular mechanism. HER2 aptamer modification promotes uptake by HER2‐expressing tumor cells, followed by acid‐responsive MNF disassembly and intracellular release of functional components. Endogenous miR‐21 and APE1 cooperatively activate the DNAzyme, enabling GLUT1 mRNA cleavage and miR‐21‐associated signaling disruption. This dual regulation suppresses glucose‐entry‐associated metabolic buffering, restores PTEN‐associated signaling and weakens copper‐handling capacity. Under this metabolically rewired state, Cu^2^^+^ and cystine inputs are more effectively converted into copper‐associated mitochondrial protein stress and disulfide‐stress‐related cytoskeletal disruption, thereby promoting tumor‐biased cell killing.

The logic‐gated DNAzyme is embedded into a Ca/Cu metal–nucleic acid framework in which the metal functions mirror the therapeutic sequence. Ca^2^^+^ supports DNAzyme‐compatible coordination and catalytic competence for the metabolic‐licensing step, whereas Cu^2^^+^ reinforces framework persistence during delivery and provides a copper‐associated stress source after intracellular release. Cystine is co‐assembled to introduce an additional disulfide‐stress input. In this architecture, HER2‐guided tumor‐cell entry, miR‐21/APE1‐dependent activation, GLUT1‐centred metabolic rewiring and copper/disulfide stress engagement are functionally coupled layers within one material entity. The effects of the metal and cystine inputs are conditioned by the extent to which GLUT1‐centred metabolic regulation weakens tumor‐cell buffering capacity and established a stress‐permissive state (Scheme 1).

Using this platform, we demonstrate a programmed therapeutic sequence from tumor‐state recognition to metabolic licensing and stress engagement. C@HGDz^2^ ^1^/CaCu suppresses GLUT1 and glycolytic signaling, is accompanied by PTEN restoration consistent with attenuation of miR‐21‐associated signaling, and remodels glucose‐derived metabolic buffering. Under this licensed state, Cu^2^^+^ and cystine inputs are associated with oxidative injury, mitochondrial dysfunction, copper‐responsive mitochondrial protein stress and disulfide‐stress‐linked cytoskeletal disruption. In an orthotopic gastric tumor model, the framework enhances tumor accumulation, preserves the designed metabolic‐rewiring‐to‐stress‐engagement sequence and suppresses tumor progression with apparent tolerability under the tested regimen. This work presents state‐gated metallo‐DNAzyme framework engineering as a materials strategy that couples tumor metabolic rewiring with coordinated stress execution, enabling the conversion of metabolic buffering into programmable therapeutic vulnerability.

## 2 Results

### 2.1 Encoding and Validating Dual‐Input Molecular Logic Within a Metallo‐DNAzyme Framework

**FIGURE 1:**
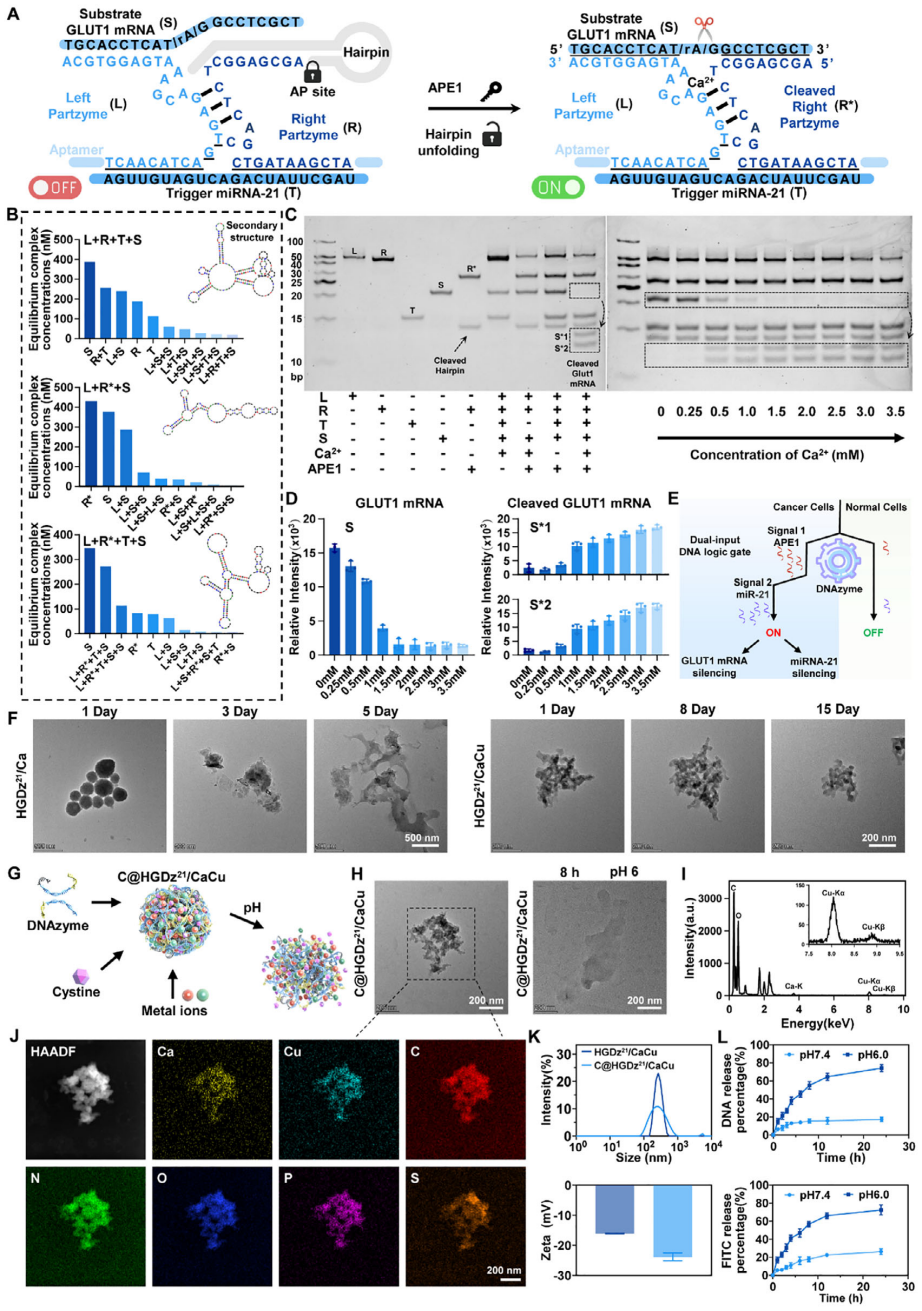
Function‐partitioned construction of a tumor‐state‐gated metallo‐DNAzyme framework. (A) Schematic illustration of the dual‐input logic‐gated DNAzyme. The catalytic core is divided into left and right partzymes, with the right partzyme sequestered in a hairpin structure containing an APE1‐responsive cleavage site. APE1‐mediated cleavage generates the unlocked right partzyme, whereas miR‐21 promotes cooperative assembly of the catalytically competent complex and functions as one of the two molecular activation inputs. Efficient cleavage of the GLUT1 RNA substrate was observed under the dual‐input condition, whereas omission of either miR‐21 or APE1 markedly reduced substrate conversion. (B) NUPACK‐predicted equilibrium complex distributions under different input conditions, supporting dual‐input‐dependent formation of the catalytically competent DNAzyme complex. (C) Polyacrylamide gel electrophoresis analysis showing input‐ and Ca^2^^+^‐dependent cleavage of the GLUT1 RNA substrate. (D) Quantification of substrate cleavage in C by ImageJ analysis. (E) In vitro truth table of dual‐input‐dependent DNAzyme cleavage, in which miR‐21 and APE1 define the ON state for GLUT1 targeting under the combined miR‐21/APE1 input condition. (F) Transmission electron microscopy images showing the time‐dependent morphology of Ca^2^^+^‐coordinated DNAzyme assemblies and Ca^2^^+^/Cu^2^^+^ co‐doped metal–nucleic acid frameworks. (G) Schematic illustration of Ca–Cu MNF construction and cystine co‐assembly. (H) TEM image of cystine‐loaded C@HGDz^2^ ^1^/CaCu nanoparticles. (I,J) Energy‐dispersive x‐ray spectroscopy spectrum (I) and elemental mapping (J) of C@HGDz^2^ ^1^/CaCu nanoparticles, confirming the presence and spatial distribution of Ca, Cu, and DNA‐associated elements. (K) Hydrodynamic size distribution and zeta potential of HGDz^2^ ^1^/Ca and C@HGDz^2^ ^1^/CaCu nanoparticles. (L) pH‐responsive release profiles of DNAzyme and FITC, used as a model small‐molecule cargo, from C@HGDz^2^ ^1^/CaCu nanoparticles at pH 7.4 and pH 6.0.

We first sought to encode tumor‐state information into DNAzyme catalysis and integrate this catalytic logic with metal‐ and cystine‐associated stress‐input capacity. To achieve this, a GLUT1‐targeting DNAzyme was engineered as a cooperative miR‐21/APE1‐gated system rather than a constitutively active catalyst. The 17E catalytic motif was divided into left and right partzymes, and the right partzyme was sequestered within an APE1‐responsive hairpin. In this design, miR‐21 promotes productive strand assembly, whereas APE1‐mediated cleavage unlocks the right partzyme. GLUT1 substrate cleavage was therefore expected to occur preferentially in a dual‐input ON state defined by miR‐21‐dependent assembly and APE1‐dependent unlocking (Figure 1A).

NUPACK analysis supported this state‐gated design. Predicted equilibrium complex distributions showed limited formation of the catalytically competent DNAzyme complex when the right partzyme remained locked or when miR‐21 was absent. In contrast, the combined presence of miR‐21 and the APE1‐unlocked right partzyme favored active‐complex formation (Figure 1B and Figures S1 and S2). These simulations indicate that neither endogenous input alone is sufficient to efficiently generate the catalytic conformation, supporting an assembly architecture consistent with AND‐gated, dual‐input‐dependent activation.

We next examined whether the predicted molecular logic could be translated into input‐dependent catalysis. Polyacrylamide gel electrophoresis showed efficient cleavage of the GLUT1 RNA substrate only when miR‐21, APE1 and Ca^2^^+^ were simultaneously present (Figure 1C). Removal of either miR‐21 or APE1 markedly reduced substrate cleavage, and quantitative analysis confirmed that robust substrate depletion was restricted to the dual‐input ON condition (Figure 1D). Additional controls clarified the metal requirement for DNAzyme activity. Ca^2^^+^ supported substrate cleavage, whereas Cu^2^^+^ alone did not produce detectable catalysis under the tested conditions. In parallel, APE1 cleaved the hairpin‐locked right partzyme in a concentration‐dependent manner, generating the unlocked strand required for productive assembly (Figure S3). These results support an input‐dependent catalytic architecture in which efficient GLUT1 substrate cleavage is restricted to the combined presence of miR‐21, APE1 and Ca^2^^+^, while separating Cu^2^^+^ from the catalytic role and reserving copper functionality for framework persistence and downstream stress engagement (Figure 1E).

Having established the sequence‐encoded catalytic logic, we next embedded the DNAzyme into a metal–nucleic acid framework designed to resolve the competing requirements of DNAzyme activity and material persistence. Ca^2^^+^ was incorporated to preserve DNAzyme‐compatible coordination and catalytic competence, whereas Cu^2^^+^ was introduced to improve framework durability and provide copper functionality after intracellular release. Transmission electron microscopy showed that Ca^2^^+^‐coordinated DNAzyme assemblies progressively lost morphological integrity during incubation, whereas Ca^2^^+^/Cu^2^^+^ co‐doped MNFs retained more stable nanoparticle structures (Figure 1F). Ratio‐dependent stability analysis further showed that Cu‐rich formulations better preserved framework morphology, whereas Ca‐rich formulations underwent progressive structural erosion (Figure S4). A Ca: Cu ratio of 1:1 was therefore selected to balance catalytic compatibility with structural persistence (Figure S5C).

Cystine was then co‐assembled during Ca–Cu MNF formation to incorporate an additional stress‐input component into the same material entity (Figure 1G). The resulting cystine‐loaded formulation, C@HGDz^2^ ^1^/CaCu, maintained compact nanoparticle morphology after cargo incorporation (Figure 1H). Energy‐dispersive x‐ray spectroscopy and elemental mapping confirmed the coexistence and spatial distribution of Ca, Cu, and DNA‐associated elements within individual nanoparticles, supporting successful construction of the co‐doped metallo‐DNAzyme framework (Figure 1I,J). Dynamic light scattering and zeta‐potential analysis further showed a nanoscale size distribution and surface charge compatible with subsequent cellular delivery (Figure 1K).

Finally, we evaluated whether the framework could release functional components under acidic conditions relevant to intracellular trafficking. C@HGDz^2^ ^1^/CaCu showed limited DNAzyme release at pH 7.4 but enhanced release at pH 6.0, indicating acid‐responsive framework disassembly (Figure 1L). FITC, used as a model small‐molecule cargo, displayed a similar pH‐dependent release profile. Together, these results show that C@HGDz^2^ ^1^/CaCu integrates four foundational design layers within one material framework: miR‐21/APE1‐gated DNAzyme catalysis, Ca^2^^+^‐supported catalytic compatibility, Cu^2^^+^‐reinforced framework persistence and cystine‐enabled stress‐input loading. This construction establishes the material basis for linking intracellular molecular computation with the subsequent sequence of tumor‐state‐dependent metabolic rewiring and stress engagement.

### 2.2 HER2‐Guided Deployment Establishes the Intracellular Access Layer of the State‐Gated Framework

After establishing the state‐gated catalytic design and metal‐ion functional partitioning of the framework, we next examined whether the integrated formulation could reach the intracellular environment required for subsequent DNAzyme activation and stress‐input engagement. HER2‐positive NCI‐N87 gastric cancer cells were incubated with Cy5‐labelled HER2‐targeted C@HGDz^2^ ^1^/CaCu or non‐targeted C@GDz^2^ ^1^/CaCu to distinguish aptamer‐assisted tumor‐cell recognition from nonspecific MNF internalization.

**FIGURE 2:**
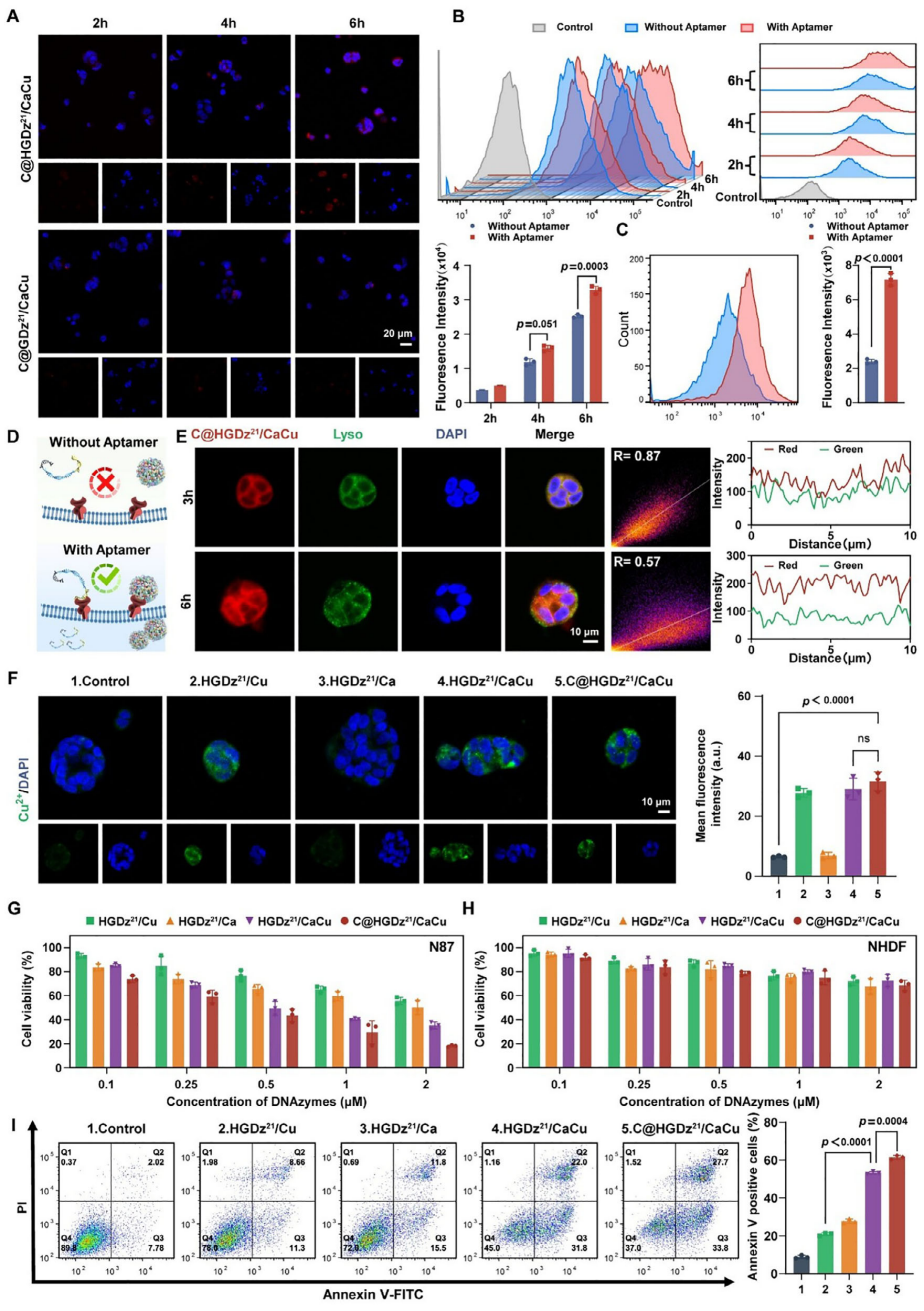
HER2‐guided spatial deployment and intracellular availability of the state‐gated metallo‐DNAzyme framework. (A) Confocal fluorescence images showing time‐dependent cellular uptake of Cy5‐labelled HER2‐targeted C@HGDz^2^ ^1^/CaCu and non‐targeted C@GDz^2^ ^1^/CaCu in HER2‐positive NCI‐N87 gastric cancer cells. (B) Flow cytometry analysis and quantification of MNF uptake, confirming enhanced internalization of HER2‐aptamer‐modified C@HGDz^2^ ^1^/CaCu compared with non‐targeted C@GDz^2^ ^1^/CaCu. (C) Flow cytometry analysis of free HGDz^2^ ^1^ and GDz^2^ ^1^ uptake, showing that HER2 aptamer modification improves molecular‐level internalization but remains less efficient than MNF‐mediated delivery. (D) Schematic illustration of HER2‐assisted endocytosis and intracellular delivery of aptamer‐functionalized MNFs. (E) Confocal lysosomal colocalization analysis of C@HGDz^2^ ^1^/CaCu over time, with ImageJ‐based quantification of fluorescence overlap coefficients, indicating progressive endo/lysosomal escape. (F) Confocal fluorescence imaging and quantification of intracellular Cu^2^^+^ accumulation in NCI‐N87 cells after treatment with different MNF formulations. (G) Viability of NCI‐N87 gastric cancer cells treated with different formulations, determined by WST‐1 assay. (H) Viability of NHDF normal fibroblasts treated under identical conditions, showing lower cytotoxicity toward normal fibroblasts under the tested conditions. (I) Flow cytometry analysis of apoptosis in NCI‐N87 cells following treatment with different formulations. Formulation nomenclature is defined in the Results section: “H” denotes HER2‐aptamer modification, “C@” denotes cystine loading, and “Ca,” “Cu,” and “CaCu” indicate the incorporated metal‐ion components.

Confocal fluorescence imaging showed time‐dependent intracellular accumulation of both formulations, but HER2‐aptamer‐modified C@HGDz^2^ ^1^/CaCu produced stronger intracellular Cy5 signals than the non‐targeted counterpart (Figure 2A). Flow cytometry confirmed this enhancement, showing higher cellular uptake of C@HGDz^2^ ^1^/CaCu relative to C@GDz^2^ ^1^/CaCu over the examined time points (Figure 2B). These results show that HER2 aptamer modification is associated with enhanced cellular uptake of the metallo‐DNAzyme framework in HER2‐expressing NCI‐N87 cells.

We then compared free DNAzyme strands with MNF‐assembled formulations to determine whether framework assembly improved intracellular deployment beyond aptamer recognition alone. Free HGDz^2^ ^1^ entered NCI‐N87 cells more efficiently than free non‐targeted GDz^2^ ^1^, showing that HER2 aptamer modification enhanced the uptake of the free DNAzyme construct under the tested conditions (Figure 2C). However, MNF assembly further increased intracellular fluorescence relative to the corresponding free DNAzyme strands. Thus, HER2 aptamer modification contributes to tumor‐cell recognition, whereas MNF assembly enhances intracellular accumulation and creates the material context for coordinated nucleic‐acid and metal deployment (Figure 2D).

Because productive GLUT1 mRNA cleavage requires cytosolic access after uptake, we next evaluated endo/lysosomal trafficking. C@HGDz^2^ ^1^/CaCu initially showed partial colocalization with lysosomal signals, consistent with endocytic internalization. This overlap decreased over time, and quantitative colocalization analysis confirmed reduced lysosomal retention at the later time point (Figure 2E). These data support gradual endo/lysosomal escape, an essential prerequisite for intracellular DNAzyme availability. Copper‐responsive fluorescence imaging further showed stronger intracellular copper‐associated signals after treatment with Cu‐containing MNF formulations than after copper‐free treatment (Figure 2F). Therefore, HER2‐guided MNF deployment was accompanied by both nucleic‐acid accessibility and detectable intracellular copper availability.

We next examined whether these deployment features translated into an initial functional response in tumor cells. In NCI‐N87 cells, HGDz^2^ ^1^/Cu produced a moderate reduction in the WST‐1 signal, indicating decreased cellular metabolic activity under the tested conditions. HGDz^2^ ^1^/Ca also reduced the WST‐1 signal, consistent with suppression of cellular metabolic activity following Ca^2^^+^‐supported DNAzyme regulation. HGDz^2^ ^1^/CaCu caused a greater reduction in the WST‐1 signal than either partial formulation, suggesting cooperation between DNAzyme‐compatible regulation and Cu‐containing framework deployment. The fully integrated cystine‐loaded formulation, C@HGDz^2^ ^1^/CaCu, produced the greatest reduction in cellular WST‐1 activity (Figure 2G), supporting the functional value of integrating targeting, DNAzyme‐compatible catalysis, Ca/Cu framework construction and cystine loading within one material system.

Under the same treatment conditions, these formulations showed lower cytotoxicity toward NHDF normal fibroblasts than toward NCI‐N87 tumor cells (Figure 2H), suggesting tumor‐cell‐biased activity within the tested in vitro setting. Flow cytometry‐based apoptosis analysis further showed that C@HGDz^2^ ^1^/CaCu increased apoptotic cell populations in NCI‐N87 cells compared with partial formulations (Figure 2I). In NCI‐N87 tumor spheroids, HER2‐targeted C@HGDz^2^ ^1^/CaCu displayed broader fluorescence distribution across the spheroid interior, whereas non‐targeted C@GDz^2^ ^1^/CaCu showed weaker and more peripheral fluorescence signals (Figure S5).

Together, these results define HER2‐guided MNF deployment as the intracellular access layer of the state‐gated framework. Aptamer modification improves tumor‐cell recognition, MNF assembly enhances intracellular accumulation, endo/lysosomal escape supports cytosolic DNAzyme availability and Cu‐containing framework deployment establishes intracellular copper availability. This access layer provides the operational prerequisites for subsequent state‐gated metabolic rewiring and stress engagement.

### 2.3 DNAzyme‐Mediated GLUT1 Regulation Establishes a Metabolically Altered Tumor‐Cell State

After establishing HER2‐guided intracellular access, we next asked whether framework deployment produced the intended metabolic‐licensing effect. The platform was designed to weaken glucose‐derived buffering before copper‐ and cystine‐associated inputs were evaluated as stress‐engaging components. We therefore first examined whether C@HGDz^2^ ^1^/CaCu could regulate the GLUT1–miR‐21 programme in NCI‐N87 tumor cells.

**FIGURE 3:**
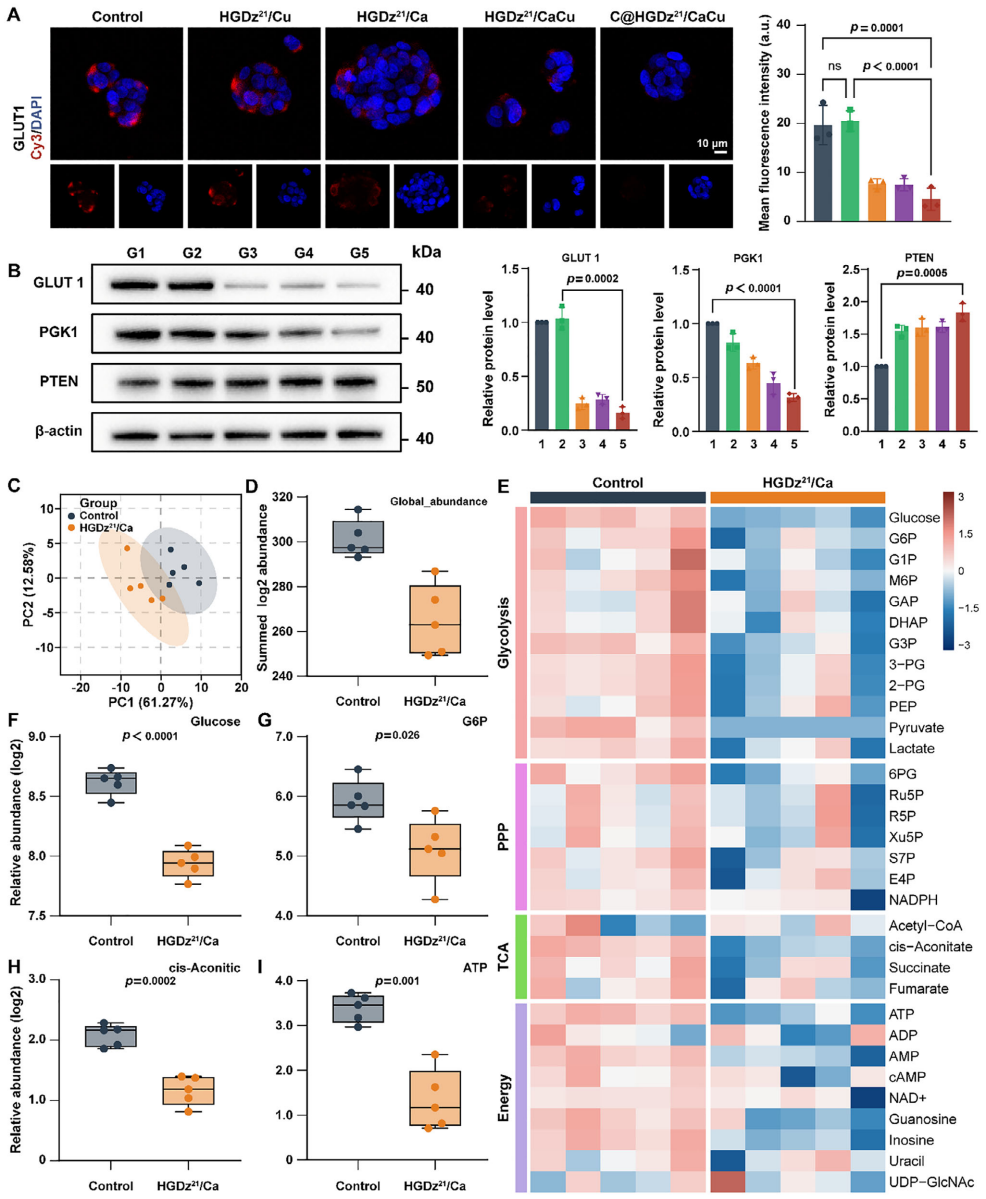
State‐gated DNAzyme catalysis rewires GLUT1–miR‐21 metabolic buffering to establish a stress‐licensing state. (A) Confocal immunofluorescence images and quantitative analysis of GLUT1 expression in NCI‐N87 cells after treatment with different formulations. GLUT1 was labelled with Cy3, and nuclei were stained with DAPI. Scale bar, 10 µm. C@HGDz^2^ ^1^/CaCu produced the strongest reduction in GLUT1 fluorescence among the treatment groups. (B) Western blot analysis and densitometric quantification of GLUT1, PGK1, and PTEN protein expression in NCI‐N87 cells after different treatments. β‐actin was used as the loading control. G1, control; G2, HGDz^2^ ^1^/Cu; G3, HGDz^2^ ^1^/Ca; G4, HGDz^2^ ^1^/CaCu; G5, C@HGDz^2^ ^1^/CaCu. DNAzyme‐active Ca‐containing formulations reduced GLUT1 and PGK1 expression, whereas PTEN expression was increased. These results demonstrate suppression of the targeted glucose‐entry and glycolysis‐associated axis accompanied by increased PTEN expression.(C) Principal component analysis of untargeted metabolomics profiles from control and HGDz^2^ ^1^/Ca‐treated NCI‐N87 cells. (D) Summed global metabolite abundance after HGDz^2^ ^1^/Ca treatment. (E) Focused heatmap of representative metabolites associated with glycolysis, the pentose phosphate pathway, TCA metabolism and cellular energy status. (F–I) Relative abundance of glucose, glucose‐6‐phosphate, cis‐aconitate and ATP. Additional metabolite‐derived pathway scores, ATP/ADP ratio and cellular energy charge are shown in Figure S6.

Confocal immunofluorescence staining showed that GLUT1 expression was reduced after treatment with DNAzyme‐containing formulations, with the strongest suppression observed in cells treated with the fully integrated C@HGDz^2^ ^1^/CaCu formulation (Figure 3A). Quantitative fluorescence analysis confirmed a marked decrease in GLUT1 signal intensity relative to control and partial‐component groups. These results demonstrate that the DNAzyme‐containing framework reduced the intended GLUT1 target after intracellular delivery to NCI‐N87 cells. Because the individual miR‐21 and APE1 inputs were not independently manipulated, this experiment demonstrates cellular target engagement rather than a complete intracellular logic truth table.

Western blot analysis provided protein‐level evidence for engagement of the targeted metabolic axis. DNAzyme‐active formulations reduced GLUT1 expression together with PGK1, a glycolysis‐associated enzyme, indicating suppression of the glucose‐entry and downstream glycolytic programme (Figure 3B). In parallel, PTEN expression increased after treatment with the fully integrated formulation. Because intracellular miR‐21 availability was not directly measured, this change is interpreted as an associated regulatory response rather than direct evidence of miR‐21 depletion. These protein‐level findings provided an orthogonal basis for interpreting the subsequent metabolomic analysis.

To characterize the metabolic state associated with DNAzyme‐mediated GLUT1 regulation while excluding direct Cu‐ and cystine‐associated stress inputs, we performed untargeted metabolomics in control and HGDz^2^ ^1^/Ca‐treated NCI‐N87 cells. This formulation retains Ca^2^^+^‐supported DNAzyme activity while excluding Cu‐containing stress input and cystine loading. Principal component analysis showed clear separation between control and HGDz^2^ ^1^/Ca‐treated cells, indicating a distinct treatment‐associated metabolic state (Figure 3C). Summed global metabolite abundance was also reduced after treatment (Figure 3D).

Focused metabolite analysis revealed coordinated decreases in glucose‐ and energy‐associated metabolite pools (Figure 3E). Glucose and glucose‐6‐phosphate were reduced after HGDz^2^ ^1^/Ca treatment, consistent with decreased glucose availability and early glycolytic input (Figure 3F,G). The abundance of cis‐aconitate and ATP was also decreased, indicating alterations in TCA‐associated metabolism and cellular energy status (Figure 3H,I). Metabolite‐derived pathway scores associated with glycolysis, TCA metabolism and nucleotide‐energy metabolism were reduced, with a decreasing trend in the PPP‐associated score (Figure S6A–D).

Importantly, HGDz^2^ ^1^/Ca treatment also reduced the ATP/ADP ratio and cellular energy charge (Figure S6H,I). Together with the decrease in ATP abundance, these changes indicate diminished adenylate energy status and impaired cellular energy buffering. Thus, the protein and metabolite data jointly support a metabolic state characterized by suppression of the intended GLUT1‐associated axis and contraction of glucose‐derived and energy‐associated metabolite pools.

Because untargeted metabolomics measures steady‐state metabolite abundance rather than metabolic flux or enzyme activity, the pathway‐level changes are interpreted as metabolic signatures consistent with reduced buffering capacity rather than direct evidence of complete pathway shutdown. The metabolomic findings indicate contraction of glucose‐derived and energy‐associated metabolite pools. Because these measurements reflect steady‐state abundance rather than mitochondrial flux, they do not establish complete suppression of mitochondrial metabolism or predict the direction of canonical cuproptosis sensitivity.

### 2.4 Metabolic‐State Conditioning Enhances Oxidative, Mitochondrial and DNA Damage‐Associated Stress Engagement

After defining GLUT1–miR‐21 regulation as the metabolic‐licensing layer of the platform, we next asked whether this rewired state was accompanied by enhanced engagement of the stress inputs delivered by the Ca/Cu framework and cystine‐loaded formulation. Because HGDz^2^ ^1^/Ca treatment reduced glucose‐derived metabolic input and energy‐associated buffering, we examined whether Cu‐ and cystine‐associated inputs more effectively induced oxidative stress, mitochondrial membrane disruption, mitochondrial ultrastructural injury and DNA damage‐associated signaling after framework treatment.

**FIGURE 4:**
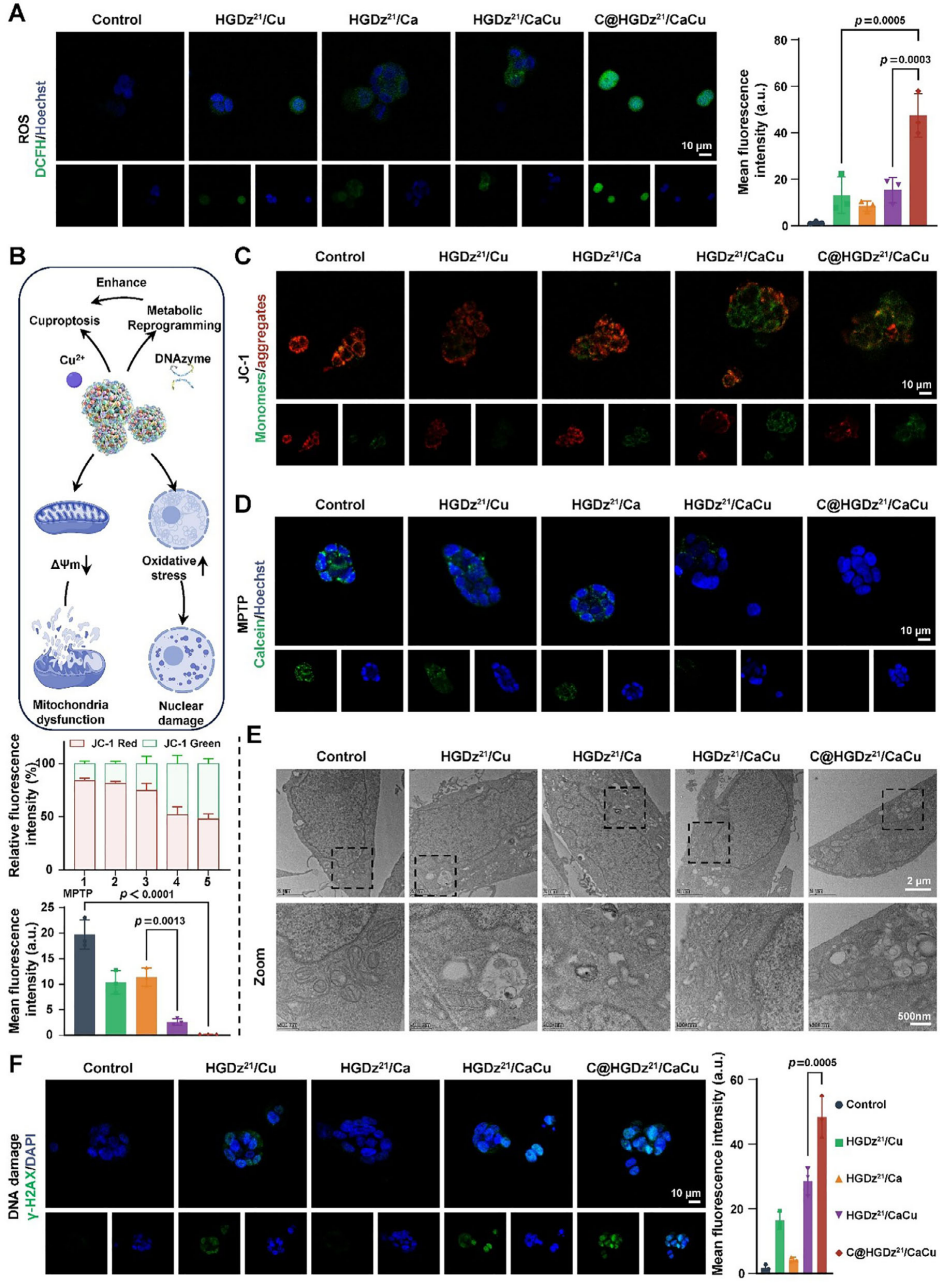
Metabolic licensing converts Cu^2^^+^ and cystine inputs into coordinated oxidative, mitochondrial, and genotoxic damage. (A) Intracellular reactive oxygen species levels in NCI‐N87 cells after treatment with different formulations, measured using the DCFH fluorescence probe. (B) Schematic illustration of the proposed stress phenotype induced by DNAzyme–MNF deployment, in which metabolic buffering disruption is associated with oxidative stress, mitochondrial dysfunction and downstream DNA damage signaling. (C) JC‐1 staining analysis of mitochondrial membrane potential, showing mitochondrial depolarization after treatment with MNF formulations. (D) Mitochondrial permeability transition pore assay, indicating impaired mitochondrial membrane homeostasis after treatment. (E) Bio‐TEM images showing mitochondrial ultrastructural changes in NCI‐N87 cells following different treatments. (F) Immunofluorescence staining of γ‐H2AX, indicating accumulation of DNA double‐strand‐break‐associated damage signals after MNF treatment.

Intracellular ROS levels were first measured using DCFH fluorescence. Partial formulations induced only limited ROS accumulation, whereas Ca/Cu‐containing MNF formulations increased intracellular ROS signals (Figure 4A). The strongest fluorescence was observed in the C@HGDz^2^ ^1^/CaCu group, indicating that cystine incorporation further strengthened oxidative stress within the Ca/Cu framework context. Together with the metabolomic evidence of reduced glucose‐derived and PPP‐associated buffering, this pattern is consistent with weakened redox‐buffering capacity after metabolic licensing.

The proposed stress‐engagement sequence is summarized in Figure 4B. In this model, DNAzyme‐mediated suppression of glucose‐entry‐associated buffering does not act as an isolated metabolic endpoint. Instead, it shifts tumor cells away from a stress‐buffered state and creates a context in which Cu‐ and cystine‐associated inputs can more effectively engage downstream stress responses.

We next examined mitochondrial membrane potential using JC‐1 staining. Control cells and cells treated with partial formulations largely retained mitochondrial polarization, as indicated by predominant red JC‐1 aggregate fluorescence. In contrast, Ca/Cu‐containing MNF formulations caused a shift toward green JC‐1 monomer fluorescence, reflecting mitochondrial depolarization (Figure 4C). This effect was most evident after C@HGDz^2^ ^1^/CaCu treatment, consistent with increased mitochondrial stress when DNAzyme‐mediated metabolic regulation, Ca/Cu framework deployment, and cystine loading were combined.

Mitochondrial permeability transition pore analysis further supported disruption of mitochondrial membrane homeostasis. Compared with control and partial‐treatment groups, MNF‐treated cells showed increased pore opening, indicating impaired mitochondrial membrane integrity (Figure 4D). Direct ultrastructural observation by bio‐TEM provided additional evidence of mitochondrial injury. Control cells displayed relatively intact mitochondrial morphology and preserved cristae, whereas cells treated with the integrated MNF platform showed mitochondrial swelling, cristae disorganization, and membrane disruption (Figure 4E). These structural changes were most pronounced in the C@HGDz^2^ ^1^/CaCu group, supporting the emergence of a mitochondrial stress phenotype after combined metabolic licensing and stress‐input deployment.

We further examined whether oxidative and mitochondrial stress engagement was accompanied by nuclear stress signaling. Immunofluorescence staining revealed increased γ‐H2AX accumulation after MNF treatment, with the strongest signal detected in the C@HGDz^2^ ^1^/CaCu group (Figure 4F). This result indicates that the treatment‐induced stress phenotype was not confined to mitochondrial perturbation, but was accompanied by DNA damage‐associated γ‐H2AX signaling.

**FIGURE 5:**
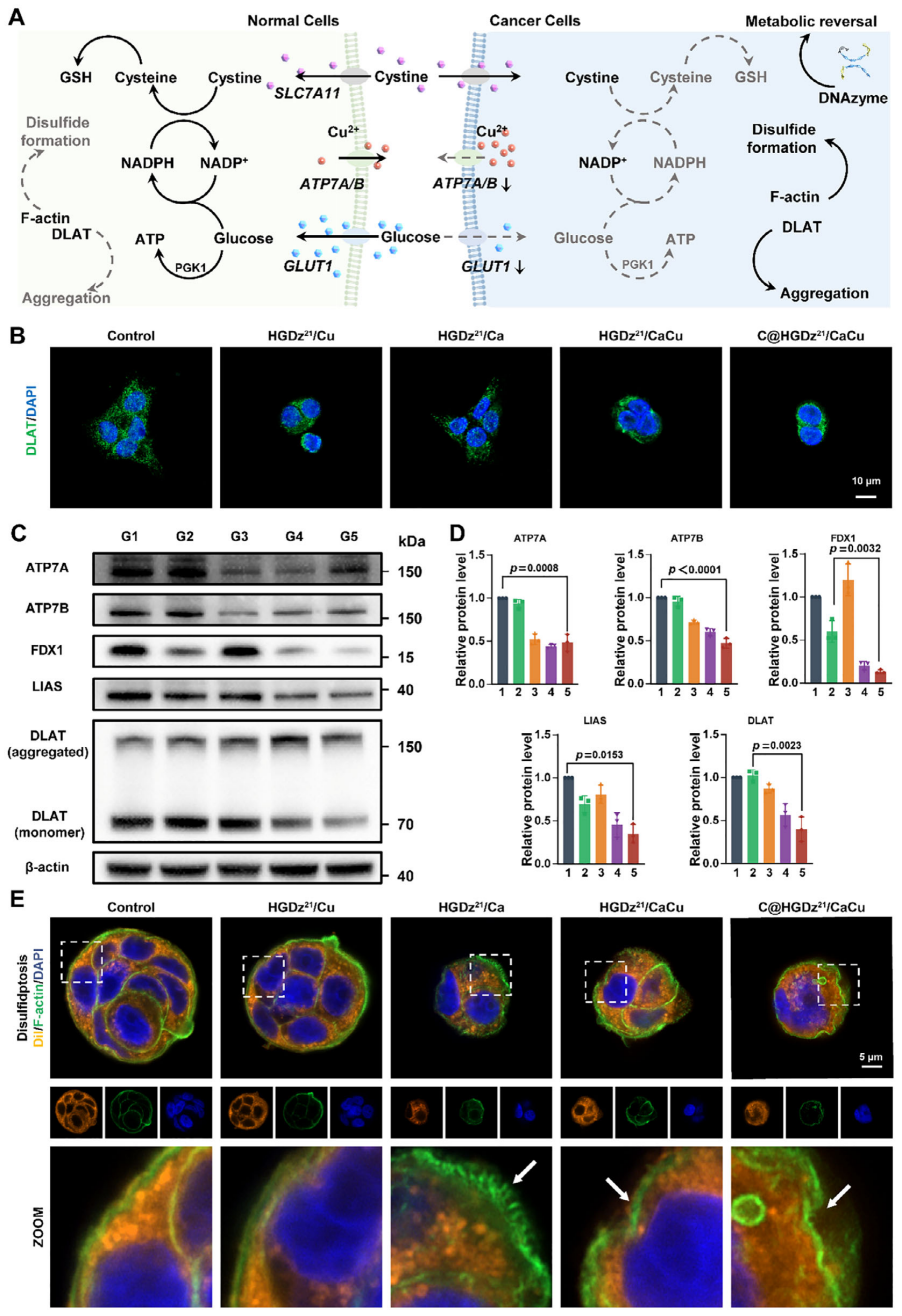
Metabolic licensing exposes copper‐handling disruption and disulfide‐stress‐linked cytoskeletal vulnerability. (A) Schematic illustration of how logic‐gated metabolic intervention increases tumor‐cell susceptibility to copper‐ and disulfide‐stress‐associated death features. Suppression of GLUT1 and miR‐21‐associated buffering weakens glycolytic ATP production, PPP‐associated redox buffering and adaptive PTEN repression, thereby reducing copper‐handling capacity and disulfide‐stress tolerance. (B) Immunofluorescence images showing DLAT redistribution and aggregation‐like puncta in NCI‐N87 cells after treatment with different formulations. (C) Western blot analysis of copper‐homeostasis‐ and cuproptosis‐associated proteins, including ATP7A, ATP7B, FDX1, LIAS and DLAT, following different treatments. (D) Densitometric quantification of protein expression levels in C, normalized to β‐actin. (E) Confocal fluorescence images of F‐actin organization in NCI‐N87 cells after treatment with different formulations. Magnified images highlight treatment‐induced actin disorganization and cytoskeletal collapse associated with disulfide‐stress vulnerability.

Together, these data show that metabolic licensing is associated with enhanced engagement of Cu‐ and cystine‐associated stress inputs. The combined increase in ROS accumulation, mitochondrial depolarization, permeability transition, ultrastructural mitochondrial injury and γ‐H2AX signaling indicates that suppression of GLUT1–miR‐21‐linked buffering shifts tumor cells toward a state more permissive to multi‐layered stress responses. This stress‐engagement layer provides the functional bridge between the metabolic rewiring established in Figure 3 and the copper‐handling and disulfide‐stress‐linked vulnerabilities examined in Figure 5.

### 2.5 Metabolic Licensing Reveals Copper‐Handling and Disulfide‐Stress‐Linked Cytoskeletal Vulnerabilities

After identifying oxidative, mitochondrial and DNA damage‐associated stress engagement, we next examined whether this response was accompanied by more specific vulnerability features related to copper handling and cystine‐associated disulfide pressure. Because the platform combines GLUT1–miR‐21 metabolic rewiring with Cu‐containing framework deployment and cystine loading, we focused on how the metabolically rewired tumor‐cell state processed these stress modalities, thereby revealing downstream stress‐response features associated with copper handling and disulfide pressure (Figure 5A).

We first analyzed DLAT distribution as a cellular readout associated with copper‐associated mitochondrial protein stress. In control cells and cells treated with partial formulations, DLAT staining remained relatively diffuse, suggesting limited aggregation‐like mitochondrial protein stress under these conditions. In contrast, Ca/Cu‐containing MNF formulations increased punctate DLAT signals, and this aggregation‐like redistribution was most evident after treatment with C@HGDz^2^ ^1^/CaCu (Figure 5B). This pattern indicates that copper‐containing framework deployment, when combined with DNAzyme‐mediated metabolic rewiring and cystine loading, promotes a mitochondrial protein‐stress phenotype consistent with increased copper‐associated vulnerability.

Western blot analysis further connected this phenotype to changes in copper‐homeostasis‐associated proteins. ATP7A and ATP7B, two copper‐export‐associated transporters, were attenuated after treatment with DNAzyme‐containing formulations, with the strongest decrease observed in the C@HGDz^2^ ^1^/CaCu group (Figure 5C,D). This pattern suggests that the treatment not only introduces a copper‐containing stress input, but also places this input into a cellular context with reduced expression of proteins linked to copper export. In parallel, C@HGDz^2^ ^1^/CaCu modulated representative copper‐associated mitochondrial stress markers, including FDX1, LIAS and DLAT. Rather than relying on a single marker, the coordinated changes in copper‐export‐associated proteins, DLAT redistribution and mitochondrial copper‐stress‐associated markers support the engagement of copper‐associated mitochondrial vulnerability under the rewired metabolic state.

We next examined intracellular thiol redox homeostasis by measuring the GSH/GSSG ratio. HGDz^2^ ^1^/Cu and HGDz^2^ ^1^/Ca each produced a moderate decrease in the GSH/GSSG ratio, consistent with copper‐associated oxidative pressure and impaired glucose‐dependent redox buffering, respectively. A greater reduction was observed after HGDz^2^ ^1^/CaCu treatment, whereas the cystine‐loaded C@HGDz^2^ ^1^/CaCu formulation produced the lowest GSH/GSSG ratio among the tested groups (Figure S5D). This progressive reduction indicates that DNAzyme‐mediated metabolic regulation, Cu‐containing framework deployment and cystine incorporation converge on intracellular thiol and redox imbalance. These findings support a coordinated copper–thiol stress environment but do not establish that copper‐associated and disulfide‐associated effects operate as chemically independent pathways.

We then assessed whether cystine incorporation was accompanied by disulfide‐stress‐linked cytoskeletal vulnerability. In control cells, F‐actin formed organized filamentous networks, whereas partial formulations induced only moderate cytoskeletal disturbance. Ca/Cu‐containing MNFs caused more evident actin disorganization, and cystine‐loaded C@HGDz^2^ ^1^/CaCu produced extensive loss of filament integrity and cytoskeletal collapse (Figure 5E). This phenotype is consistent with increased susceptibility to disulfide‐stress‐linked cytoskeletal disruption when glucose‐derived energy and redox buffering are weakened.

Collectively, these results refine the stress‐engagement phenotype into two downstream vulnerability patterns. First, attenuation of copper‐export‐associated proteins, DLAT aggregation‐like redistribution, and modulation of mitochondrial copper‐stress‐associated markers indicate enhanced copper‐associated mitochondrial protein stress. Second, cystine loading is accompanied by marked F‐actin disruption, indicating increased cytoskeletal sensitivity to disulfide‐stress‐associated pressure. These findings support a model in which C@HGDz^2^ ^1^/CaCu does not simply deliver copper and cystine as independent toxic inputs. Instead, it integrates GLUT1‐centred metabolic regulation with copper‐ and cystine‐associated inputs, allowing these stress components to act within a metabolically rewired cellular state. Although Cu‐containing framework treatment increased intracellular copper‐associated fluorescence and induced mitochondrial dysfunction together with DLAT redistribution, the present study did not directly resolve the subcellular localization of copper. These findings are therefore interpreted as a copper‐associated mitochondrial stress phenotype rather than direct evidence of mitochondrial Cu^2^^+^ accumulation.

### 2.6 HER2‐Guided Systemic Deployment Translates the State‐Gated Framework Into Orthotopic Tumor Suppression

**FIGURE 6:**
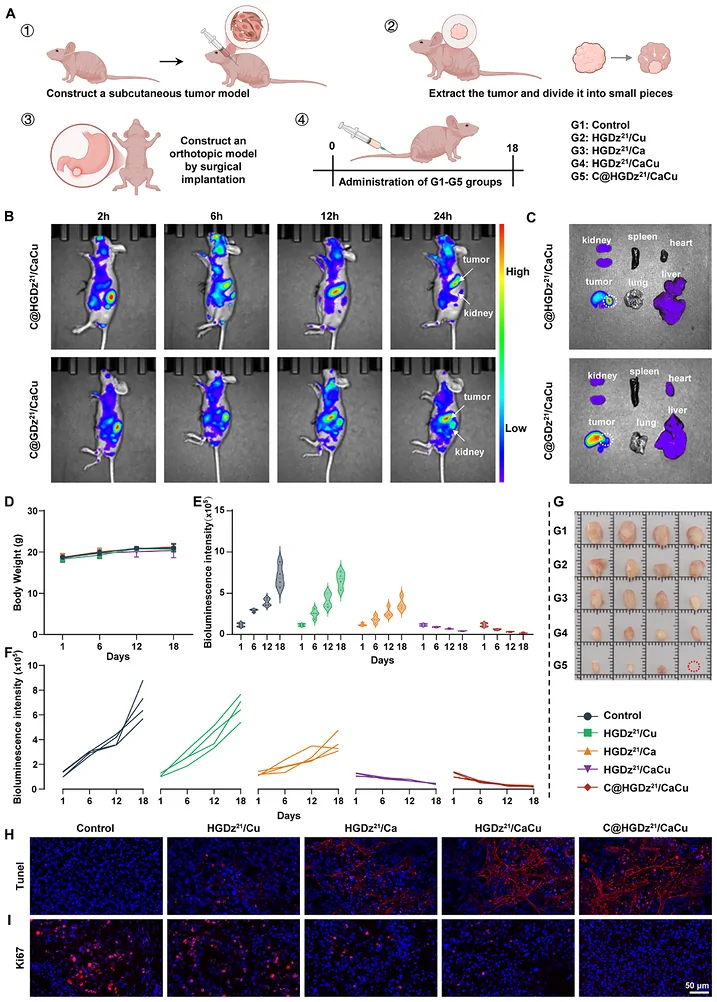
HER2‐guided orthotopic deployment and antitumor efficacy of the state‐gated metallo‐DNAzyme framework. (A) Schematic illustration of the establishment and treatment of the orthotopic gastric tumor model. NCI‐N87 tumor fragments were surgically implanted into the gastric wall, and tumor‐bearing mice were assigned to five treatment groups: G1, control; G2, HGDz^2^ ^1^/Cu; G3, HGDz^2^ ^1^/Ca; G4, HGDz^2^ ^1^/CaCu; and G5, C@HGDz^2^ ^1^/CaCu. (B) Time‐dependent in vivo fluorescence imaging of mice after intravenous injection of Cy5‐labelled HER2‐targeted C@HGDz^2^ ^1^/CaCu or non‐targeted C@GDz^2^ ^1^/CaCu, showing enhanced tumor accumulation of the HER2‐aptamer‐modified formulation. (C) Ex vivo fluorescence images of tumors and major organs collected 24 h after injection, comparing biodistribution of HER2‐targeted and non‐targeted MNFs. (D) Body‐weight changes during the treatment period. (E) Distribution of tumor‐associated bioluminescence intensities on days 1, 6, 12 and 18. (F) Individual bioluminescence trajectories showing tumor progression or suppression in each mouse. (G) Representative photographs of excised tumors collected at the endpoint. (H,I) Representative TUNEL (H) and Ki67 (I) staining of orthotopic tumor sections after treatment.

After establishing the in vitro sequence of intracellular access, metabolic licensing and stress engagement, we next evaluated whether the integrated framework could achieve tumor enrichment and antitumor activity after systemic administration. An orthotopic gastric tumor model was established by surgically implanting NCI‐N87 tumor fragments into the gastric wall, providing an anatomically relevant setting for assessing in vivo delivery and therapeutic response. Tumor‐bearing mice were assigned to five treatment groups: control, HGDz^2^ ^1^/Cu, HGDz^2^ ^1^/Ca, HGDz^2^ ^1^/CaCu and C@HGDz^2^ ^1^/CaCu (Figure 6A). This group design allowed the in vivo contributions of copper input, Ca^2^^+^‐supported DNAzyme regulation, Ca/Cu framework integration and cystine‐loaded full formulation to be compared within the same orthotopic tumor setting.

We first examined whether HER2 aptamer modification enhanced tumor enrichment after intravenous administration. Whole‐body fluorescence imaging showed time‐dependent accumulation of both Cy5‐labelled HER2‐targeted C@HGDz^2^ ^1^/CaCu and non‐targeted C@GDz^2^ ^1^/CaCu in tumor‐bearing mice. The HER2‐aptamer‐modified formulation generated stronger tumor‐associated fluorescence over time than the non‐targeted formulation (Figure 6B). Ex vivo imaging of tumors and major organs collected 24 h after injection further supported this pattern. Compared with non‐targeted C@GDz^2^ ^1^/CaCu, HER2‐targeted C@HGDz^2^ ^1^/CaCu produced higher fluorescence in tumor tissues, whereas fluorescence in major organs reflected broader systemic distribution after intravenous administration (Figure 6C). These results support aptamer‐associated enhancement of orthotopic tumor enrichment by the metallo‐DNAzyme framework.

We then assessed whether this systemic deployment was accompanied by suppression of orthotopic tumor progression. Longitudinal bioluminescence measurements showed progressive signal expansion in control mice and in mice treated with partial formulations, whereas C@HGDz^2^ ^1^/CaCu restrained tumor‐associated signal expansion during the treatment period (Figure 6E). Individual tumor‐signal trajectories further showed more consistent signal control across mice receiving the fully integrated formulation (Figure 6F). Endpoint tumor photographs were consistent with the imaging results, with tumors from the C@HGDz^2^ ^1^/CaCu group appearing smaller than those from the other groups (Figure 6G).

Supporting tissue staining further corroborated the therapeutic response. Tumor sections from C@HGDz^2^ ^1^/CaCu‐treated mice showed increased TUNEL‐positive signals and reduced Ki67 staining compared with control and partial‐treatment groups, indicating increased tumor‐cell apoptosis and reduced proliferation in vivo (Figure 6H,I). These findings are consistent with the longitudinal optical imaging data and support the antitumor activity of the fully integrated state‐gated framework in the orthotopic tumor model.

We performed a preliminary tolerability assessment based on longitudinal body‐weight monitoring and histological examination of major organs. Body weights remained stable across treatment groups during the treatment period (Figure 6D), and H&E staining of the heart, liver, spleen, lung and kidney revealed no apparent histopathological abnormalities under the tested regimen (Figure S8). Together, these observations support preliminary systemic tolerability under the tested conditions.

Together, these in vivo results show that HER2‐guided systemic deployment of C@HGDz^2^ ^1^/CaCu supports orthotopic tumor enrichment, suppresses tumor‐associated optical progression and shows apparent tolerability under the tested regimen. The fully integrated formulation outperformed partial formulations, supporting the therapeutic value of combining tumor‐state‐gated DNAzyme regulation, Ca/Cu framework deployment and cystine‐enabled stress‐input loading within one material system.

### 2.7 Orthotopic Tumors Show Coordinated Metabolic Rewiring and Copper‐Associated Stress Engagement After State‐Gated Framework Therapy

**FIGURE 7:**
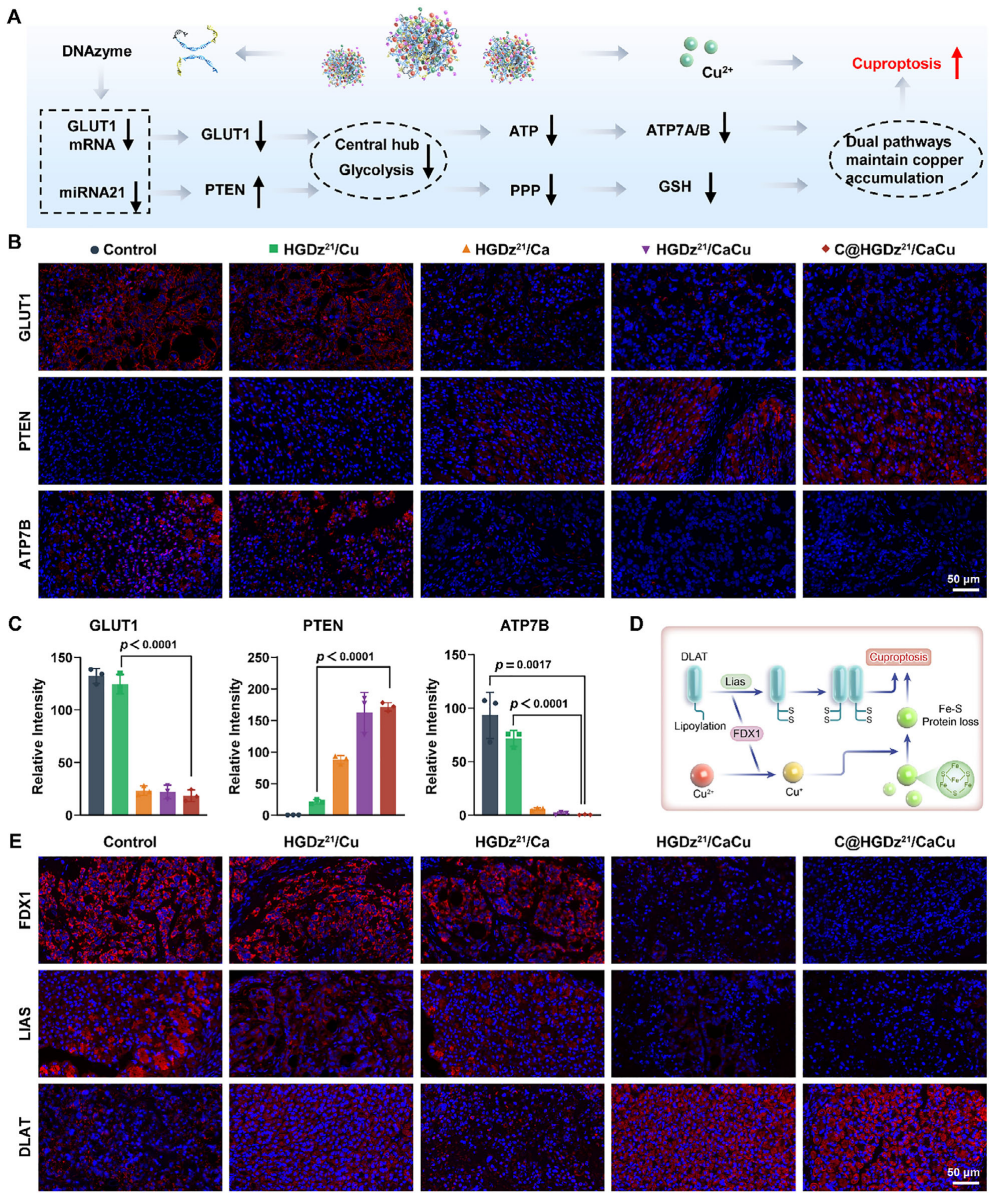
In vivo preservation of metabolic rewiring and copper‐associated stress engagement in orthotopic tumors. (A) Schematic illustration of the proposed in vivo sequence by which state‐gated framework therapy suppresses glucose‐dependent metabolic buffering, restores PTEN‐associated regulation and reduces copper‐export‐associated buffering, thereby increasing susceptibility to copper‐associated mitochondrial protein stress. (B) Representative immunofluorescence images of GLUT1, PTEN and ATP7B expression in tumor sections collected after different treatments. (C) Quantification of immunofluorescence intensities in B, showing coordinated GLUT1 and ATP7B downregulation together with increased PTEN expression after C@HGDz^2^ ^1^/CaCu treatment. (D) Schematic illustration of cuproptosis‐associated mitochondrial stress, highlighting copper‐dependent mitochondrial protein stress, lipoylated‐protein aggregation‐like changes and disruption of iron–sulfur cluster‐related mitochondrial homeostasis. (E) Immunofluorescence staining of cuproptosis‐associated proteins, including FDX1, LIAS and DLAT, in tumor sections after different treatments. C@HGDz^2^ ^1^/CaCu treatment induced marked alterations in these markers and promoted DLAT aggregation‐like redistribution, supporting enhanced copper‐associated mitochondrial stress in vivo.

After confirming orthotopic tumor enrichment and tumor‐signal suppression in vivo, we next examined whether the designed metabolic‐rewiring‐to‐stress‐engagement sequence was preserved in tumor tissues. Because the platform was designed to suppress glucose‐entry‐associated buffering before enhancing copper‐associated stress susceptibility, we first assessed GLUT1 and PTEN as tissue‐level markers of DNAzyme‐mediated metabolic regulation and miR‐21‐associated regulatory restoration. ATP7B was included as a copper‐export‐associated protein to evaluate whether this metabolic transition was accompanied by changes in copper‐handling‐associated buffering (Figure 7A).

Immunofluorescence staining of orthotopic tumor sections showed that C@HGDz^2^ ^1^/CaCu reduced GLUT1 expression compared with control and partial‐treatment groups (Figure 7B). This result indicates that the DNAzyme framework retained its ability to engage the glucose‐entry target in vivo. In parallel, PTEN expression increased after treatment with the fully integrated formulation, consistent with attenuation of miR‐21‐associated repression in tumor tissues. These coordinated changes support in vivo remodeling of the GLUT1–miR‐21/PTEN regulatory programme and suggest weakened glucose‐dependent metabolic buffering within orthotopic tumors.

Within this metabolically rewired context, ATP7B expression was also decreased in the C@HGDz^2^ ^1^/CaCu group (Figure 7B). Rather than proving copper‐export function directly, this reduction indicates attenuation of a copper‐export‐associated protein in treated tumors. Quantitative analysis confirmed decreased GLUT1 and ATP7B fluorescence together with increased PTEN signal intensity after C@HGDz^2^ ^1^/CaCu treatment (Figure 7C). This tissue‐level pattern mirrors the in vitro regulatory response and supports the view that orthotopic tumor suppression is accompanied by metabolic‐state remodeling and altered copper‐homeostasis‐associated protein expression.

We further examined whether this tissue‐level metabolic transition was accompanied by copper‐associated mitochondrial stress features. Because C@HGDz^2^ ^1^/CaCu supplies a Cu‐containing framework component while weakening metabolic buffering, we assessed representative proteins linked to copper‐associated mitochondrial stress, including FDX1, LIAS and DLAT (Figure 7D). Immunofluorescence staining revealed treatment‐associated alterations in these markers after C@HGDz^2^ ^1^/CaCu administration (Figure 7E). Tumor sections from the fully integrated formulation group showed stronger copper‐stress‐associated mitochondrial features and a more punctate, aggregation‐like DLAT redistribution pattern, consistent with enhanced mitochondrial protein‐stress engagement under conditions of copper input and reduced metabolic protection.

Collectively, these tissue‐level data support preservation of the intended functional coupling in vivo. C@HGDz^2^ ^1^/CaCu suppresses the GLUT1‐associated metabolic entry point, increases PTEN‐associated regulatory signaling, attenuates ATP7B expression and enhances copper‐associated mitochondrial stress features in orthotopic tumor tissues. These findings do not assign the therapeutic response to a single isolated death pathway. Instead, they support a model in which state‐gated metabolic rewiring reduces tumor buffering capacity and places copper‐associated inputs into a tissue context more permissive to mitochondrial stress engagement. This in vivo mechanistic layer closes the sequence from framework deployment to metabolic licensing, stress engagement and orthotopic tumor suppression.

## 3 Conclusions

In summary, this work establishes a design principle for state‐conditioned stress‐executing materials. Instead of treating therapeutic efficacy as a direct consequence of stress delivery, we show that the executability of delivered stress can be programmed by the metabolic state of recipient tumor cells. C@HGDz^2^ ^1^/CaCu embodies this principle by integrating three material functions within one framework. Sequence‐encoded miR‐21/APE1 logic produces dual‐input‐dependent GLUT1 substrate cleavage under cell‐free conditions, while the assembled framework supports GLUT1 regulation after delivery to tumor cells. Ca/Cu functional partitioning separates DNAzyme‐compatible catalysis from framework persistence and copper‐associated stress‐input capacity. Cystine loading further introduces a second stress input whose impact is conditioned by prior metabolic rewiring.

Through this architecture, the framework first weakens GLUT1‐dependent metabolic buffering and then places metal‐ and disulfide‐associated inputs into a cellular context more permissive to oxidative, mitochondrial and cytoskeletal stress engagement. In an orthotopic gastric tumor model, this functionally coupled architecture translated into tumor enrichment, tumor suppression and preservation of the metabolic‐rewiring‐to‐stress‐engagement pattern under the tested regimen. Conceptually, this study shifts the role of therapeutic materials from passive stress carriers to active regulators of tumor stress permissiveness. By linking molecular‐state computation, metal‐ion role partitioning and metabolic licensing, state‐gated metallo‐DNAzyme frameworks provide a blueprint for designing materials that determine not only where stress is delivered, but also when and how it becomes biologically effective.

## Author Contributions

X.M. conceived the project, designed the overall experimental strategy, led the experimental work, and performed the main experiments. L.H. and X.W. contributed substantially to cellular experiments, material synthesis, data acquisition, and data analysis. Y.W. assisted with cellular experiments. C.L. and J.Y. contributed to material synthesis and related characterization. X.M., L.H. and X.W. analysed and interpreted the data. X.M. wrote the initial manuscript draft. H.Z. provided conceptual guidance, writing supervision and critical revision of the manuscript. W.S., X.S. and H.Z. supervised the project and provided funding support. All authors discussed the results, revised the manuscript, and approved the final version.

## Conflicts of Interest

The authors declare no conflicts of interest.

## Supporting information




**Supporting File**: advs77520‐sup‐0001‐SuppMat.docx.

## Data Availability

The data that support the findings of this study are available on request from the corresponding author. The data are not publicly available due to privacy or ethical restrictions.
