# Supplementary material for: A State‐Gated Metallo‐DNAzyme Framework Programs Tumor Stress Vulnerability Through Metabolic Licensing
Source: Adv Sci (Weinh). 2026 Sep 8:e77520. Online ahead of print. doi: 10.1002/advs.77520 (PMC13554373; doi:10.1002/advs.77520)
Supplement: Supplementary file 1 [file ADVS-9999-e77520-s001.docx]

**Supporting Information**

**A State-Gated Metallo-DNAzyme Framework Programs Tumour Stress Vulnerability through Metabolic Licensing**

Xiaodong Ma^1,2&^, Lingyun He^1,3&^, Xinru Wang^1,2&^, Yuanyuan Wei^1,3^, Chengcheng Li^2,4^, Jiaqi Yan^2,4^, Weijian Sun^1,3^*, Xian Shen^1,3^*, Hongbo Zhang^1,2,4^*

1 NHC Key Laboratory of Clinical Nutrition and Intervention, The First Affiliated Hospital of Wenzhou Medical University, Wenzhou, China

2 Pharmaceutical Sciences Laboratory, Faculty of Science and Engineering, Åbo Akademi University, Turku, Finland

3 Zhejiang Key Laboratory of Intelligent Cancer Biomarker Discovery and Translation, The First Affiliated Hospital of Wenzhou Medical University, Wenzhou, China

4 Turku Bioscience Centre, University of Turku and Åbo Akademi University, Turku, Finland

^&^These authors contributed equally: Xiaodong Ma, Lingyun He, Xinru Wang

Corresponding Author E-mails: Hongbo Zhang: [hongbo.zhang@abo.fi](mailto:hongbo.zhang@abo.fi); Xian Shen: shenxian@wmu.edu.cn; Weijian Sun: fame198288@126.com.

**Materials and Methods**

**Reagents and oligonucleotides**

All oligonucleotides used in this study were synthesized and HPLC-purified by Sangon Biotechnology. The sequences of the DNAzyme strands, miR-21-responsive module, APE1-responsive module and GLUT1 substrate are provided in Supplementary Table 1. Calcium and copper salts, cystine, acrylamide/bis-acrylamide solution, electrophoresis buffers, GelRed, RIPA buffer, BCA protein assay reagents, protease inhibitors, fluorescent probes and routine chemical reagents were obtained from commercial suppliers and used as received. Primary antibodies against GLUT1, DLAT, LIAS and PGK1 were obtained from Proteintech; FDX1 from Abcam; ATP7A from Abmart; ATP7B and β-actin from Affinity Biosciences; γ-H2AX from Beyotime; and PTEN from HuaBio. All antibodies were used according to the manufacturers’ recommendations.

**Cell culture**

NCI-N87 human gastric cancer cells were cultured in RPMI-1640 medium supplemented with 10% fetal bovine serum at 37 °C in a humidified incubator containing 5% CO₂. Normal human dermal fibroblasts were cultured in DMEM supplemented with 10% fetal bovine serum and 1% penicillin–streptomycin under the same conditions. Cells were passaged regularly before reaching full confluence.

**DNAzyme design and NUPACK analysis**

The logic-gated DNAzyme was designed by splitting the catalytic DNAzyme motif into left and right partzymes. The right partzyme was sequestered in an APE1-responsive hairpin structure, whereas miR-21 was used to promote assembly of the catalytically active complex and to function as a therapeutic sink. The assembled DNAzyme was designed to cleave a GLUT1 RNA substrate.

Secondary structures and equilibrium complex distributions of different strand combinations were predicted using the NUPACK Web App. The analysed systems included combinations of the left partzyme, locked right partzyme, APE1-unlocked right partzyme, miR-21 trigger strand and GLUT1 substrate. The predicted structures and assembly probabilities were used to evaluate whether productive DNAzyme formation required both miR-21-mediated strand organization and APE1-mediated unlocking.

**In vitro DNAzyme cleavage assay**

DNAzyme cleavage reactions were performed by mixing the indicated DNAzyme strands with the GLUT1 RNA substrate in the presence or absence of miR-21, APE1, Ca²⁺ or Cu²⁺. The reactions were incubated at 37 °C and then analysed by polyacrylamide gel electrophoresis. For gel analysis, samples were mixed with loading buffer and loaded onto 20% polyacrylamide gels. Electrophoresis was performed at 90 V for approximately 2 h. Gels were stained with GelRed and imaged using a gel documentation system. Band intensities were quantified using ImageJ, and substrate cleavage was calculated from the decrease in intact substrate signal. APE1-mediated unlocking of the hairpin-locked right partzyme was evaluated by incubating the locked strand with different concentrations of APE1, followed by gel electrophoresis analysis. Metal specificity was assessed by comparing Ca²⁺- and Cu²⁺-containing reaction conditions.

**Preparation of Ca–Cu MNFs and cystine-loaded MNFs**

Metal–nucleic acid frameworks were prepared through metal-ion-mediated self-assembly. Briefly, 2 µL of DNAzyme-containing oligonucleotides (1 mM) were mixed with CaCl₂ and/or CuCl₂ stock solutions (1 M each) to obtain a total reaction volume of 200 µL. For Ca–Cu MNFs, Ca²⁺ and Cu²⁺ were added at a molar ratio of 1:1. Ca-only and Cu-only formulations were prepared using the corresponding metal ion under otherwise identical conditions. The mixtures were incubated at ambient temperature to allow particle formation. For cystine-loaded MNFs, cystine was added during the Ca–Cu-mediated assembly process. HER2-targeted C@HGDz²¹/CaCu and non-targeted C@GDz²¹/CaCu were prepared using the corresponding aptamer-modified and non-targeted DNAzyme strands, respectively. After assembly, the nanoparticles were collected by centrifugation, washed and resuspended in PBS before use.

**Nanoparticle characterization and release assays**

Nanoparticle morphology was examined by transmission electron microscopy. Hydrodynamic size and zeta potential were measured by dynamic light scattering. Elemental composition and distribution were analysed by energy-dispersive X-ray spectroscopy and elemental mapping. Time-dependent stability of Ca-, Cu- and Ca–Cu-containing MNFs was evaluated by incubating the nanoparticles under the indicated conditions and monitoring morphological changes by TEM.

For pH-responsive release assays, C@HGDz²¹/CaCu nanoparticles were incubated in buffers at pH 7.4 or pH 6.0. At predetermined time points, released DNAzyme or FITC, used as a model small-molecule cargo, was quantified from the supernatant. Release percentages were calculated relative to the total loaded amount.

**Cellular uptake, lysosomal escape and intracellular Cu²⁺ detection**

For cellular uptake analysis, NCI-N87 cells were seeded in confocal dishes and incubated overnight. Cells were then treated with Cy5-labelled free DNAzyme or Cy5-labelled MNF formulations for the indicated times. After treatment, cells were washed, fixed, counterstained with DAPI and imaged by confocal microscopy. For flow cytometry analysis, treated cells were collected, washed and analysed in the Cy5 channel. For lysosomal escape analysis, cells were incubated with Cy5-labelled C@HGDz²¹/CaCu for the indicated times and then stained with LysoTracker Green. Colocalization between Cy5-labelled MNFs and lysosomes was analysed using ImageJ. Intracellular Cu²⁺ availability was assessed using a copper-responsive fluorescent probe. After treatment with Cu-containing or copper-free formulations, cells were incubated with the probe according to the manufacturer’s instructions and imaged by confocal microscopy. Fluorescence intensities were quantified using ImageJ.

**Cell viability, apoptosis and tumour spheroid penetration**

For cell viability assays, NCI-N87 cells or NHDFs were seeded in 96-well plates at 5,000 cells per well and cultured overnight. Cells were treated with different formulations for 24 h. WST-1 reagent was then added to the medium, and cells were incubated for an additional 3 h. Absorbance at 450 nm was measured using a microplate reader, and viability was normalized to untreated controls.

Cell apoptosis was assessed by flow cytometry after treatment with different formulations. Cells were collected, stained using an apoptosis detection kit according to the manufacturer’s instructions and analysed by flow cytometry. For tumour spheroid penetration assays, NCI-N87 tumour spheroids were incubated with Cy5-labelled HER2-targeted or non-targeted MNFs. Spheroids were washed and imaged by confocal microscopy. Penetration was evaluated from the distribution of Cy5 fluorescence across the spheroid.

**Western blotting**

After treatment, cells were lysed in RIPA buffer supplemented with protease inhibitors and EDTA. Protein concentrations were determined using a BCA protein assay kit. Equal amounts of protein were separated by SDS–PAGE and transferred onto PVDF membranes. Membranes were blocked, incubated with primary antibodies against the indicated proteins and then incubated with HRP-conjugated secondary antibodies. Protein bands were visualized using a chemiluminescence imaging system. β-actin was used as the loading control. Band intensities were quantified using ImageJ.

**Immunofluorescence and confocal microscopy**

For cellular immunofluorescence, cells were seeded in confocal dishes or on coverslips and treated with different formulations. After treatment, cells were fixed with 4% paraformaldehyde, permeabilized when required, blocked and incubated with primary antibodies against the indicated targets, including GLUT1, γ-H2AX and DLAT. After washing, cells were incubated with fluorescent secondary antibodies and counterstained with DAPI. F-actin was visualized using fluorescent phalloidin according to the manufacturer’s instructions. For tissue immunofluorescence, tumour sections were processed by standard staining procedures and incubated with antibodies against the indicated markers, including GLUT1, PTEN, ATP7B, FDX1, LIAS, DLAT and Ki67. Nuclei were counterstained with DAPI. All confocal images within each experiment were acquired using identical imaging settings. Fluorescence intensities, colocalization coefficients and puncta-like signal distributions were quantified using ImageJ from randomly selected fields or predefined regions of interest.

**Untargeted metabolomics**

NCI-N87 cells were treated with HGDz²¹/Ca or control medium for the indicated time. Cells were washed with cold PBS, and metabolites were extracted using a pre-cooled organic extraction solvent. Samples were centrifuged to remove insoluble material, and supernatants were collected for untargeted LC–MS analysis. Quality-control samples were prepared by pooling aliquots from individual samples. Raw metabolomics data were processed for peak detection, alignment, signal normalization and metabolite annotation using standard metabolomics workflows. Missing or non-detected metabolite abundance values were imputed using one-half of the minimum detected abundance for the corresponding metabolite before log₂ transformation. Principal component analysis, global metabolite abundance analysis, pathway activity analysis and focused metabolite analysis were performed using normalized metabolite abundance values. For heatmap visualization, log₂-transformed metabolite abundance values were further normalized by row-wise z-score transformation across samples. Representative metabolites associated with glycolysis, the pentose phosphate pathway, TCA metabolism and energy metabolism were selected for focused analysis.

**ROS, mitochondrial and cytoskeletal stress assays**

Intracellular ROS levels were measured using DCFH-DA staining. After treatment with different formulations, cells were incubated with DCFH-DA according to the manufacturer’s instructions, washed and imaged by confocal microscopy. Mitochondrial membrane potential was assessed using JC-1 staining. Treated cells were incubated with JC-1 working solution, and red JC-1 aggregate fluorescence and green JC-1 monomer fluorescence were imaged by confocal microscopy. Mitochondrial permeability transition pore opening was evaluated using a calcein-AM/CoCl₂-based assay. After staining, cells were imaged by confocal microscopy, and mitochondrial fluorescence changes were analysed. For F-actin analysis, treated cells were fixed, permeabilized and stained with fluorescent phalloidin. Cytoskeletal organization was evaluated by confocal microscopy.

**Bio-TEM analysis**

For ultrastructural analysis, treated NCI-N87 cells were collected, fixed, embedded and sectioned using standard procedures for transmission electron microscopy. Ultrathin sections were mounted on copper grids, stained with uranyl acetate and lead citrate, and examined by transmission electron microscopy. Mitochondrial morphology, cristae organization and membrane integrity were evaluated from representative images.

**Orthotopic gastric tumour model and treatments**

All animal experiments were approved by the Institutional Animal Care and Use Committee at the Zhejiang Center of Laboratory Animals. Orthotopic gastric tumours were established by surgically implanting luciferase-labelled NCI-N87 tumour fragments into the gastric wall of mice. After tumour establishment, mice were assigned to different treatment groups, including control, HGDz²¹/Cu, HGDz²¹/Ca, HGDz²¹/CaCu and C@HGDz²¹/CaCu. Tumour progression was monitored by longitudinal optical imaging. Body weight was recorded during the treatment period. At the endpoint, mice were euthanized, and tumours and major organs were collected for ex vivo imaging, histology and immunofluorescence analysis.

**In vivo biodistribution and imaging**

For biodistribution studies, orthotopic tumour-bearing mice were intravenously injected with Cy5-labelled HER2-targeted C@HGDz²¹/CaCu or non-targeted C@GDz²¹/CaCu. Whole-body fluorescence imaging was performed at the indicated time points after injection. At 24 h after injection, mice were euthanized, and tumours and major organs were collected for ex vivo fluorescence imaging. Fluorescence intensities were quantified by region-of-interest analysis using identical imaging settings. For therapeutic monitoring, orthotopic tumours expressing luciferase were imaged after luciferin administration. Tumour-associated optical signals were quantified from defined regions of interest and plotted over time.

**Histology, TUNEL staining and tumour immunofluorescence**

Excised tumours and major organs were fixed, embedded and sectioned using standard histological procedures. Major organ sections were stained with haematoxylin and eosin for histopathological evaluation. Tumour apoptosis was assessed by TUNEL staining according to the manufacturer’s instructions. Tumour-cell proliferation was assessed by Ki67 immunofluorescence staining. For mechanistic tissue analysis, tumour sections were stained for GLUT1, PTEN, ATP7B, FDX1, LIAS and DLAT. Images were acquired by confocal microscopy, and fluorescence intensities were quantified using ImageJ.

**Statistics and reproducibility**

Data are presented as mean ± s.d. unless otherwise indicated. Statistical analyses were performed using GraphPad Prism. Comparisons between two independent groups were performed using two-tailed unpaired Student’s *t*-tests. Comparisons among multiple independent groups were performed using one-way ANOVA followed by multiplicity-adjusted pairwise comparisons in GraphPad Prism. *P* values are indicated in the figures or figure legends. For cell experiments, *n* represents biologically independent samples unless otherwise stated. For animal experiments, *n* represents individual mice.

**Supplementary Information**

***Supplementary Table 1: Oligonucleotide sequences***

| **Name** | **Sequence (5'→3')** |
| --- | --- |
| Trigger miRNA-21 | TAGCTTATCAGACTGATGTTGA |
| Substrate Glut1 mRNA | GGACCCTGCACCTCAT/rA/GGCCTCGCTGGCATG |
| Left partzyme | GCAGCGGTGTGGGGGCAGCGGTGTGGGGGCAGCGGTGTGGGGTCAACATCAGTGAGACGAAATGAGGTGCA |
| Right partzyme | AGAGCCTCGCTTCGCCACATAAGGCG/idSp/AGCGAGGCTCTCAGCCTGATAAGCTAGCAGCGGTGTGGGG |

***Supplementary Fig. 1 NUPACK-predicted secondary structures and free energies of different DNA strand combinations.*** *Representative secondary structures of different DNA strand combinations involved in logic-gated DNAzyme assembly were predicted using the NUPACK Web App. The analysed combinations include L+S, R*+S, L+R*+S, L+S+T, L+R*+S+T and L+R+S+T, where L and R denote the left and right partzymes, R* denotes the nuclease-unlocked form of R, T denotes the miR-21 trigger strand and S denotes the GLUT1 mRNA substrate. The predicted minimum free energy values are shown below each structure. These simulations provide structural support for the strand hybridisation landscape underlying the dual-input DNAzyme design.*

***Supplementary Fig. 2 Schematic interpretation of predicted assembly routes for logic-gated DNAzyme activation.*** *Proposed assembly routes of different DNA strand systems based on NUPACK-predicted hybridisation behaviour. In the L+R+T+S system, sequestration of R within a hairpin structure limits productive assembly, resulting in low or negligible probability of forming the catalytically competent L+R+S+T complex. In the L+R*+S system, the absence of the miR-21 trigger strand T still disfavors productive complex formation, despite nuclease-mediated unlocking of R. In contrast, in the L+R*+T+S system, release of R* together with the presence of T markedly favours cooperative assembly of the active DNAzyme complex. L and R denote the left and right partzymes, R* denotes the nuclease-unlocked form of R, T denotes the miR-21 trigger strand, and S denotes the GLUT1 mRNA substrate.*

***Supplementary Fig. 3 Metal specificity and APE1-mediated unlocking of the logic-gated DNAzyme.*** *(A) Gel electrophoresis showing that increasing Cu²⁺ concentrations failed to induce detectable cleavage of the GLUT1 mRNA substrate (S), confirming that Cu²⁺ alone cannot support DNAzyme catalysis. (B) Gel electrophoresis showing APE1 concentration-dependent cleavage of the hairpin-locked R strand, as evidenced by the appearance of R* and cleaved hairpin fragments.*

***Supplementary Fig. 4 Effect of Ca:Cu ratio on MNF stability over time.*** *TEM images of MNFs assembled with Cu only, Ca:Cu = 1:2 and Ca:Cu = 2:1 after incubation for 1, 8 and 15 days. Cu-containing MNFs with higher Cu²⁺ ratios exhibited improved morphological stability, whereas Ca-rich MNFs gradually lost structural integrity. At day 15, no intact MNF structures were observed in the Ca:Cu = 2:1 group, suggesting extensive degradation. These observations support the role of Cu²⁺ in reinforcing MNF stability.*

***Supplementary Fig. 5 Penetration of HER2-targeted and non-targeted MNFs in tumour spheroids.*** *Confocal z-stack images of NCI-N87 tumour spheroids after incubation with Cy5-labelled MNFs. (A) HER2 aptamer-modified C@HGDz^21^/CaCu showed broad fluorescence distribution across the spheroid interior, indicating enhanced three-dimensional penetration. (B) Non-targeted C@GDz^21^/CaCu exhibited weaker and more peripheral fluorescence distribution, suggesting limited penetration without HER2 aptamer modification. Red fluorescence represents Cy5-labelled MNFs, and grey indicates bright-field spheroid morphology.* *(C) Effect of the Ca:Cu ratio on the functional activity of metallo-DNAzyme frameworks. Cell viability of NCI-N87 cells after treatment with MNFs assembled at Ca:Cu ratios of 9:1, 7:3, 1:1, 3:7 and 1:9 under equivalent DNAzyme dosing conditions. (D) Intracellular GSH/GSSG balance after treatment with different metallo-DNAzyme formulations.*

***Supplementary Fig. 6 HGDz^21^/Ca treatment suppresses glycolysis-associated metabolic activity and energy buffering in NCI-N87 cells.*** *(A–D) Untargeted metabolomics-based pathway activity scores showing changes in glycolysis, pentose phosphate pathway (PPP), tricarboxylic acid cycle (TCA) and nucleotide-energy metabolism after HGDz^21^/Ca treatment compared with control cells. HGDz^21^/Ca reduced glycolysis, TCA and nucleotide-energy pathway activity, with a decreasing trend in PPP activity. (E–I) Relative abundance of representative metabolites and energy-related indices, including 6-phosphogluconate (6PG), lactate, AMP, ATP/ADP ratio and energy charge. HGDz^21^/Ca treatment decreased 6PG and AMP levels and reduced ATP/ADP ratio and energy charge, indicating impaired glucose-derived metabolic buffering and cellular energy homeostasis after DNAzyme-mediated GLUT1 regulation. Data are shown as box plots with individual data points; n = 5 biologically independent samples per group. FDR-adjusted values are shown for pathway activity scores, and P values are shown for individual metabolites.*

***Supplementary Fig. 7 Extended metabolomic heatmap showing the broader metabolite profile underlying the focused metabolic analysis in Fig. 3.*** *Heatmap showing the relative abundance of annotated metabolites detected in control and HGDz²¹/Ca-treated NCI-N87 cells by untargeted metabolomics. Columns represent individual biological samples, and rows represent metabolites grouped by metabolic class, including carbohydrate metabolism, sugar phosphates, phosphorylated metabolites, organic acids, nucleotide metabolism, amino acid metabolism, amino acid derivatives, coenzymes and vitamins, and LPE. The colour scale indicates row-normalized relative abundance, with red representing higher and blue representing lower metabolite levels. The metabolites highlighted in Fig. 3E represent a focused subset of this broader dataset, selected for their direct relevance to glycolysis, the pentose phosphate pathway, TCA metabolism and cellular energy buffering. This extended heatmap provides the full metabolic context for the pathway-level changes shown in the main figure and shows that HGDz²¹/Ca treatment broadly remodels glucose-derived, nucleotide-energy and amino-acid-associated metabolic networks.*

***Supplementary Fig. 8 In vivo preliminary biosafety evaluation of MNF formulations.*** *Representative H&E staining images of major organs, including the heart, liver, spleen, lung and kidney, collected after treatment. No apparent histopathological abnormalities were observed across treatment groups under the tested conditions, suggesting favourable preliminary systemic tolerability of the MNF formulations.*
